# The burden of premature mortality among older adults: a population-based study in Malaysia

**DOI:** 10.1186/s12889-022-13608-9

**Published:** 2022-06-14

**Authors:** Yee Mang Chan, Shubash Shander Ganapathy, LeeAnn Tan, Nazirah Alias, Nur Hamizah Nasaruddin, Wan-Fei Khaw

**Affiliations:** Institute for Public Health, National Institutes of Health, Ministry of Health Malaysia, Blok B5&B6, Kompleks NIH, No 1, Jalan Setia Murni U13/52, Seksyen U13, Setia Alam, Shah Alam, Selangor 40170 Malaysia

**Keywords:** Premature mortality, Older adults, Burden of disease, Malaysia, Years of life lost, YLL

## Abstract

**Background:**

The populations of many countries—including Malaysia—are rapidly growing older, causing a shift in leading causes of disease and death. In such rapidly ageing populations, it is critical to monitor trends in burden of disease and health of older adults by identifying the leading causes of premature mortality and measuring years of life lost (YLL) to these. The objective of this study, therefore, is to describe the burden (quantified by YLL) associated with major causes of premature mortality among older adults in Malaysia in 2019.

**Methods:**

All deaths that occurred in older adults aged 60 and above in Malaysia in the year 2019 were included in this study. YLL was calculated by summing the number of deaths for the disease category at 5-year age intervals, multiplied by the remaining life expectancy for the specific age and sex group. Both life expectancy and mortality data were obtained from the Department of Statistics Malaysia.

**Results:**

In 2019, older adults accounted for 67.4% of total deaths in Malaysia (117,102 out of 173,746). The total number of YLL among older adults in Malaysia in 2019 was estimated at 1.36 million YLL, accounting for 39.6% of the total YLL (3.44 million) lost to all premature deaths in that year. The major causes of premature mortality among older adults were ischaemic heart disease (29.5%) followed by cerebrovascular disease (stroke) (20.8%), lower respiratory infections (15.9%), diabetes mellitus (8.1%) and trachea, bronchus and lung cancers (5.0%).

**Conclusions:**

Non-communicable diseases (NCD) remained the largest contributor to premature mortality among older adults in Malaysia. Implementation of population-level NCD health promotion programmes, screening programmes among high-risk groups and holistic intervention programmes among populations living with NCD are critical in reducing the overall burden of premature mortality.

## Background

As life expectancy increases, many countries must contend with rapidly aging populations. Globally, it is estimated that the number of older adults will more than double between 2017 to 2050 [1]. Older populations in less developed countries are growing at a faster rate than in more developed regions [2]. Malaysia is experiencing a similar demographic transition where the number of older adults is anticipated to increase from 7 to 14% over the next 23 years, from 2020 to 2042 [3].

The rapidly growing number of older adults has caused a shift in leading causes of disease and death. As people get older, they develop a number of complex health conditions known as geriatric syndromes, which are frequently the result of multiple underlying factors, such as frailty, urinary incontinence, falls, delirium, and pressure ulcers [4]. The proportion of people aged 65 years and older increased globally from 6.1% to 8.8%, and the number of global deaths increased by 9 million, between 1990 and 2017. Compared to 1990, 12 million additional global deaths in 2017 were associated with population ageing, corresponding to 27.9% of total global deaths [5]. This phenomenon has been observed in high-income, upper-middle income, as well as lower-middle income countries alike [5].

The disability-adjusted life year (DALY) is a popular metric for assessing population health [6]. DALY has two components; the fatal component is represented by years of life lost [YLL] and the non-fatal component by years lived with disability (YLD) [7]. Some studies assessed the burden of specific diseases, such as COVID-19 [8–10], while some only assessed YLL to estimate the burden of premature mortality [11–13]. Given Malaysia’s rapidly ageing population, it is critical to monitor the burden of disease and health of older adults in the country. Identifying major causes of premature mortality and common contributors to YLL among older adults will provide important evidence for health policy development and planning. Therefore, this study aimed to describe the burden (quantified by YLL) associated with major causes of premature mortality among older adults in Malaysia in 2019.

## Methods

### Classification of diseases

GBD has classified disease and injury causes into three broad categories: Group I consisting of communicable diseases and maternal, perinatal and nutritional disorders, Group II comprising non-communicable diseases and Group III being all injuries [14]. The three broad categories are further subdivided into 22 cause categories of diseases, which are then classified into specific diseases. The list of specific diseases in this study have been modified based on the epidemiological significance of particular diseases in Malaysia as well as in consultation with various public health experts; as a result, 112 specific diseases are included.

### Estimation of premature mortality

The calculation of premature mortality in terms of years of life lost (YLL) among older adults in Malaysia in 2019 was based on methods developed by Murray and Lopez for the Global Burden of Disease Study (GBD) [15]. YLL was calculated by summing the number of deaths for the disease category at 5-year age intervals, multiplied by the remaining life expectancy for the specific age and sex group. As such, the formula is as follows:

$$\mathbf{YLL}\boldsymbol(\mathbf c\boldsymbol,\mathbf s\boldsymbol,\mathbf a\boldsymbol,\mathbf t\boldsymbol)\boldsymbol\;\boldsymbol=\boldsymbol\;\mathbf N\boldsymbol(\mathbf c\boldsymbol,\mathbf s\boldsymbol,\mathbf a\boldsymbol,\mathbf t\boldsymbol)\boldsymbol\;\mathbf x\boldsymbol\;\mathbf{LE}\boldsymbol(\mathbf s\boldsymbol,\mathbf a\boldsymbol,\mathbf t\boldsymbol)$$

Where:

N (c,s,a,t) is the number of deaths due to cause c; for the given age-group, a; and sex, s; in year t

LE (s,a,t) is the remaining life expectancy for the given age-group, a; and sex, s; in year t

Mortality data in Malaysia is first collected via the vital registration system by the National Registration Department Malaysia. Subsequently, the mortality data will be sent to the Department of Statistics Malaysia (DOSM) to assign ICD-10 codes for each death. This study obtained data on number of deaths by sex, age and cause of death with corresponding ICD-10 codes from DOSM.

In Malaysia, there are two systems for death certification, namely medically certified deaths (MCD) and non-medically certified deaths (NMCD). MCD are deaths that occur in health facilities and certified by an attending physician. On the other hand, NMCD are deaths that occur outside of health facilities and reported to the local police station by the next of kin. NMCDs are not as reliable as MCD for reporting causes of death and may result in underestimation of causes of death. In recent years, the practice of verbal autopsy, usually conducted by the nearest health district office on NMCD, has been implemented in order to reduce the number of ill-defined causes of death [16]. In order to improve the data quality of NMCD, causes of death for NMCD is estimated by applying cause-specific mortality fractions (CSMF) derived from verbal autopsy [16].

Life expectancy data were obtained from life tables generated by DOSM based on mortality statistics over a three-year period and mid-year population estimates for the year 2019. The observed mean age at death in the age interval and the life expectancy numbers at the exact age corresponding to the age interval were used to calculate the age and sex-specific mean life expectancy.

In this study, YLL were computed without age-weighting or discounting. Outliers and illogical data were identified and corrected during the data cleaning process. Ill-defined causes of death were reclassified and redistributed where needed according to the general approach proposed by Murray and Lopez [6]. CSMF derived from verbal autopsy data were applied on NMCD data for quality improvement. All deaths that occurred in older adults aged 60 and above in Malaysia in the year 2019 were included in this study. Throughout the conduct of the study, the tenets of the Declaration of Helsinki were followed and compliance to the Malaysian Guideline for Good Clinical Practice was ensured. Ethical approval for the study was obtained from the Medical Research and Ethics Committee (MREC), Ministry of Health Malaysia (NMRR-21–1355-60,662). All data were analysed using Microsoft Office Excel version 2010.

## Results

### Years of life lost by broad cause group

In 2019, older adults contributed to 67.4% of total deaths in Malaysia (117,102 out of 173,746). Older males and females contributed to 36.0% and 31.4% of total deaths in 2019, respectively. The total number of YLL among older adults in Malaysia in 2019 was estimated at 1.36 million, 39.6% out of the total YLL (3.44 million) lost to all premature deaths in that year. Older males accounted for 52.7% while older females contributed 47.3% YLL out of the total YLL among older adults in Malaysia in 2019.

Table 1 presents the YLL by sex and broad cause group among older adults in Malaysia in 2019. The YLL rate per 1000 population among older adults was 404.5, with a rate of 436.0 YLL per 1000 population in males and 374.5 YLL per 1000 population in females. Older males had higher YLL rates per 1000 population across all three broad groups compared to older females. Group II conditions accounted for the highest YLL among older adults (83.5%), followed by Group I conditions (13.7%) and Group III conditions (2.8%).

**Table 1:** Years of life lost (YLL) by sex and broad cause group among older adults, Malaysia, 2019

Broad cause group	Total			Males			Females		
	**YLL**	**%**	**Rate/** **1000**	**YLL**	**%**	**Rate/** **1000**	**YLL**	**%**	**Rate/** **1000**
Group I	186,349	13.7	55.4	94,191	13.1	57.3	92,158	14.3	53.6
Group II	1,135,065	83.5	337.7	596,542	83.3	363.1	538,523	83.7	313.4
Group III	38,395	2.8	11.4	25,582	3.6	15.6	12,813	2.0	7.5
**Total**	**1,359,809**	**100**	**404.5**	**716,315**	**100**	**436.0**	**643,494**	**100**	**374.5**

Group I: Communicable, maternal, perinatal and nutritional conditions

Group II: Non-communicable diseases

Group III: Injuries

**Fig. 1:**
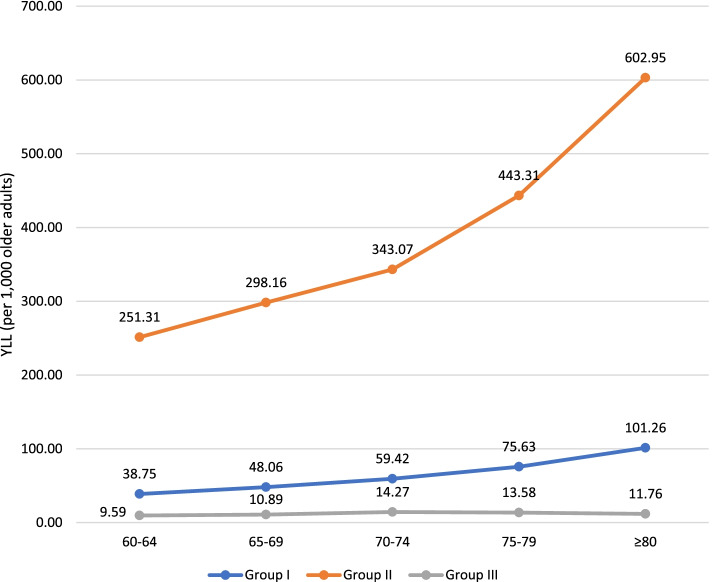
Years of life lost (YLL) rates by three broad cause groups and age-group among older adults, Malaysia, 2019

**Fig. 2:**
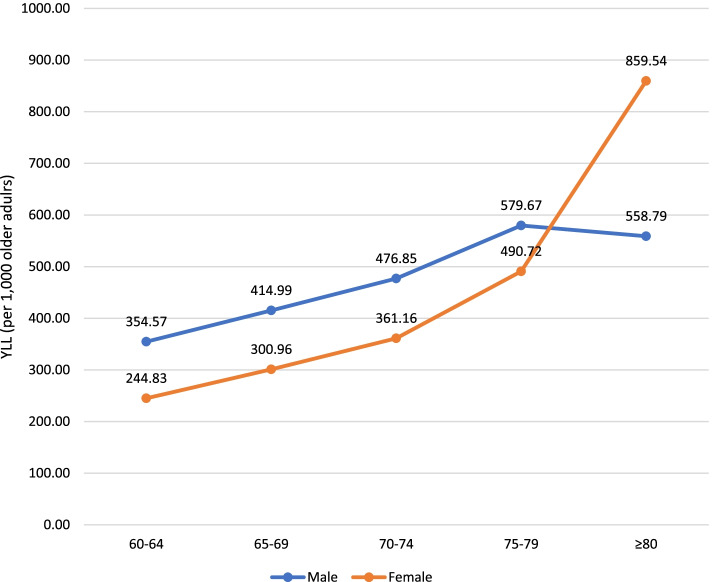
Years of life lost (YLL) rates by sex and age-group among older adults, Malaysia, 2019

The number of YLL was observed to increase with older age-groups for Group I and Group II conditions. The steepest increase of YLL in Group I was seen between the 75–79 and ≥ 80 age-groups. In contrast, YLL contributed by Group III conditions showed an increasing trend between the 60–64 and 70–74 age-groups, but decreased from the 70–74 to the ≥ 80 age group (Fig. 1). In all age groups with the exception of ≥ 80, YLL rates were higher in males than females (Fig. 2).

### Years of life lost by cause categories

Table 2 presents YLL by sex and cause categories among older adults in Malaysia 2019. Cardiovascular and circulatory diseases and malignant neoplasms were the two most common cause categories contributing to the overall YLL. These two cause categories accounted for more than half of the total YLL among older adults in 2019, with cardiovascular and circulatory diseases contributing as much as 41% of YLL and malignant neoplasms accounting for 17.8%. The third highest cause category was respiratory infections (11.7%), followed by respiratory diseases (6.1%) and diabetes mellitus (5.9%).

**Table 2:** Years of life lost (YLL) by sex and cause categories among older adults, Malaysia, 2019

Cause category	Total				Males				Females			
	**Rank**	**YLL**	**%**	**Rate/** **1000**	**Rank**	**YLL**	**%**	**Rate/** **1000**	**Rank**	**YLL**	**%**	**Rate/** **1000**
Cardiovascular and circulatory diseases	1	556,779	41.0	165.6	1	300,664	42.0	183.0	1	256,116	39.8	149.0
Malignant neoplasms	2	241,409	17.8	71.8	2	129,187	18.0	78.6	2	112,222	17.5	65.3
Respiratory infections	3	158,394	11.7	47.1	3	76,647	10.7	46.7	3	81,747	12.7	47.6
Respiratory diseases	4	83,544	6.1	24.9	4	46,859	6.5	28.5	5	36,685	5.7	21.4
Diabetes mellitus	5	80,490	5.9	23.9	5	36,932	5.2	22.5	4	43,558	6.8	25.4
Genitourinary Diseases	6	52,455	3.9	15.6	7	22,710	3.2	13.8	6	29,745	4.6	17.3
Digestive diseases	7	45,832	3.4	13.6	8	26,555	3.7	16.2	7	19,277	3.0	11.2
Unintentional injuries	8	38,006	2.8	11.3	6	25,377	3.5	15.4	10	12,629	2.0	7.4
Infectious diseases	9	27,824	2.0	8.3	9	17,479	2.4	10.6	8	10,345	1.6	6.0
Skin diseases	10	21,470	1.6	6.4	11	8707	1.2	5.3	11	12,763	2.0	7.4
Endocrine, blood, and immune disorders	11	20,884	1.5	6.2	10	9022	1.3	5.5	9	11,862	1.8	6.9
Neurological conditions	12	14,059	1.0	4.2	12	6770	1.0	4.1	12	7289	1.1	4.2
Musculoskeletal diseases	13	10,506	0.8	3.1	13	5456	0.8	3.3	13	5050	0.8	2.9
Benign Neoplasms	14	7200	0.5	2.1	14	3553	0.5	2.2	14	3647	0.6	2.1
Intentional injuries	15	389	0.0	0.1	15	205	0.0	0.1	15	184	0.0	0.1
Mental and behavioural disorders	16	167	0.0	0.1	18	60	0.0	0.0	16	107	0.0	0.1
Oral conditions	17	133	0.0	0.0	16	31	0.0	0.0	17	102	0.0	0.1
Nutritional deficiency	18	131	0.0	0.0	17	65	0.0	0.0	18	66	0.0	0.0
Sense organ diseases	19	71	0.0	0.0	19	21	0.0	0.0	19	50	0.0	0.0
Congenital anomalies	20	65	0.0	0.0	20	15	0.0	0.0	20	50	0.0	0.0
Maternal conditions	21	0	0.0	0.0	21	0	0.0	0.0	21	0	0.0	0.0
Neonatal conditions	22	0	0.0	0.0	22	0	0.0	0.0	22	0	0.0	0.0
**Total**		**1,359,809**	**100**	**404.5**		**716,315**	**100**	**436.0**		**643,494**	**100**	**374.5**

Older males share the similar top five cause categories of YLL as the overall YLL. On the other hand, the top three cause categories of YLL among older females were identical to the overall YLL, followed by diabetes mellitus as the fourth leading cause category of YLL and respiratory diseases as the fifth leading cause category of YLL.

### Years of life lost by specific diseases

Table 3 shows the top ten specific diseases that contributed the most YLL in Malaysia in 2019—ranked overall as well as by sex. Overall, the specific disease which caused the highest amount of YLL among older adults was ischaemic heart disease (29.5%), followed by cerebrovascular disease (stroke) (20.8%), lower respiratory infections (15.9%), diabetes mellitus (8.1%) and trachea, bronchus and lung cancers (5.0%). The top four leading causes of YLL in both sexes were ischaemic heart disease, cerebrovascular diseases (stroke), lower respiratory infections and diabetes mellitus. Trachea, bronchus and lung cancers was the fifth leading cause of death in males and other circulatory diseases was the fifth leading cause of death in females.

**Table 3:** Top ten leading causes of Years of life lost (YLL) by sex and specific diseases, Malaysia, 2019

Specific diseases	Total				Males^a^				Females^a^			
	**Rank**	**YLL**	**%**	**Rate/** **1000**	**Rank**	**YLL**	**%**	**Rate/** **1000**	**Rank**	**YLL**	**%**	**Rate/** **1000**
Ischaemic heart disease	1	292,504	29.5	87.0	1	167,674	31.8	102.1	1	124,830	26.8	72.6
Cerebrovascular disease (stroke)	2	206,713	20.8	61.5	2	102,768	19.5	62.6	2	103,945	22.3	60.5
Lower respiratory infections	3	158,253	15.9	47.1	3	76,574	14.5	46.6	3	81,679	17.5	47.5
Diabetes mellitus	4	80,490	8.1	23.9	4	36,932	7.0	22.5	4	43,558	9.4	25.4
Trachea, bronchus and lung cancers	5	49,347	5.0	14.7	5	33,981	6.5	20.7	9	15,366	3.3	8.9
Other respiratory diseases	6	48,078	4.8	14.3	6	25,250	4.8	15.4	6	22,828	4.9	13.3
Other circulatory diseases	7	47,746	4.8	14.2	7	24,781	4.7	15.1	5	22,965	4.9	13.4
Colon and rectum cancers	8	40,227	4.1	12.0	8	23,274	4.4	14.2	8	16,953	3.6	9.9
Nephritis and nephrosis	9	34,858	3.5	10.4	10	16,070	3.1	9.8	7	18,788	4.0	10.9
Other digestive diseases	10	34,727	3.5	10.3	9	19,921	3.8	12.1	10	14,806	3.2	8.6
**Total**		**992,943**		**295.4**		**527,225**		**320.9**		**465,718**		**271.0**

^a^Specific diseases that did not fall within the top ten contributors to overall YLL have been excluded from these sex-specific rankings

## Discussion

This study describes the burden (quantified by YLL) associated with major causes of premature mortality among older adults in Malaysia in 2019. In 2019, deaths in older adults contributed to 67.4% of total deaths, with males accounting for 36% and females accounting for 31.4%. The percentage of total deaths in older adults in Malaysia in 2019 is slightly higher compared to year 2014 (64.5%) [17]. Furthermore, the overall YLL among older adults has risen from 1,091,729 in 2014 to 1,359,809 in 2019 [17]. This could be attributed to an increase in total deaths. Findings of this study revealed that non-communicable diseases (NCD) remained major contributors to premature mortality among older adults, which is consistent with findings reported in year 2014 [17].

NCDs are responsible for 71% of deaths worldwide—equivalent to 41 million deaths. Out of that, low- and middle-income countries account for 77% of all NCD deaths [18]. In addition, NCD mortality rates in certain countries in the Asia–Pacific regions are 3–8 times higher in the older age-group (50–69 years) compared to the younger-age group (15–49 years) [19]. In Malaysia, older adults were found to have a high prevalence of self-reported diabetes (27.7%,), hypertension (51.1%), and hypercholesterolemia (41.8%) [20]. Given the high prevalence of NCD among older adults, there is a pressing need to strengthen the existing NCD prevention and management action plans and initiatives to reduce the burden of mortality among older adults in Malaysia.

Our study showed that ischaemic heart disease was the leading disease-specific contributor of YLL among older adults in Malaysia in 2019, followed by cerebrovascular disease (stroke) and lower respiratory infections. This is consistent with findings from 2014, when the top three leading disease-specific contributors of YLL among older adults in Malaysia were cerebrovascular disease (stroke), ischaemic heart disease, and chronic obstructive pulmonary disease [17]. Ischaemic heart disease and stroke are the two largest contributors to disease-specific deaths globally between 1990 to 2017 [5]. Similarly in Malaysia, ischaemic heart disease and stroke have caused the highest number of deaths between 2009 and 2014 [21].

When comparing the 2019 estimates to those from 2014, we observed several changes in the top ten specific causes of YLL among older adults in Malaysia. Chronic obstructive pulmonary disease, road traffic injuries and liver cancers have all dropped out of the top ten specific causes of YLL [17]. On the other hand, other specific causes of YLL that remained in the top ten list included cerebrovascular diseases (stroke), ischaemic heart disease, lower respiratory infections, diabetes mellitus, trachea, bronchus, and lung cancers, nephritis and nephrosis, and colon and rectum cancers [17]. This demonstrates that the major disease-specific contributors of YLL in Malaysian older adults have remained mostly consistent over time.

Cardiovascular and circulatory diseases, as well as malignant neoplasms, were the top two leading cause categories contributing to YLL among both male and female older adults in Malaysia in 2019. In 2014, these two categories were also the top two YLL cause categories among older adults in Malaysia [17]. Similarly, cardiovascular diseases and malignant neoplasms were also reported to be the leading causes of YLL in both males and females in Poland, Spain and Hong Kong [11–13].

According to our study findings, cardiovascular and circulatory diseases contributed to the highest YLL among older adults Malaysia in 2019 in both sexes, mainly due to ischaemic heart disease and cerebrovascular disease. Cardiovascular diseases account for half of all NCD deaths worldwide, with deaths from low- and middle-income countries making up almost 70% of these [22]. It has been shown that hypertension, hyperlipidaemia, diabetes, and smoking are key risk factors for heart disease and stroke [23]. Focusing on heart and stroke disease prevention by targeting these risk factors will ideally result in a reduction of disease burden and a lower risk of mortality from these diseases. In Malaysia, the prevalence of hypertension, diabetes, and hypercholesterolaemia has been on the rise from 1996 to 2015 [24]. Hypertension and hypercholesterolaemia affected more than half of older adults in Malaysia in 2015, whereas more than a third of older adults in Malaysia were living with diabetes. The increasing prevalence of risk factors will invariably increase the incidence of ischaemic heart disease and cerebrovascular disease, leading to an increased risk of death. Various initiatives and action plans have been developed and implemented by the government to reduce the burden of NCD in Malaysia, including the National Strategic Plan for NCD (NSP-NCD) 2016–2025 [25]. The implementation of this strategic plan is in line with other initiatives and action plans, for instance the National Plan of Action for Nutrition of Malaysia (NPANM) III 2016–2025 and the National Strategic Plan for Active Living 2016–2025 to ensure the effectiveness of implementation.

The second highest cause category contributing to YLL among older adults in Malaysia in 2019 is malignant neoplasms. In both sexes, trachea, bronchus and lung cancers, as well as colon and rectum cancers were amongst the commonest causes of death. A nationwide study reported that cancer symptom and risk factor awareness in Malaysia was relatively low across the nation compared to high income countries [26]. Thus, the Ministry of Health (MOH) Malaysia has taken various initiatives and steps to reduce the increasing burden of cancer morbidity and mortality, including the National Strategic Plan for Cancer Control Programme (NSPCCP) 2016 – 2020. The aim of NSPCCP 2016–2020 was to reduce the negative impact of cancer by decreasing cancer morbidity and mortality, as well as to improve the quality of life for cancer patients and their families [27]. As an example of cancer prevention initiatives, the MOH Malaysia has funded the provision and delivery of a nationwide opportunistic colorectal cancer screening in health facilities nationwide, with robust screening, follow-up, and data collection methods [28].

A strength of this study is the quantification of major causes of premature mortality in terms of YLL among older adults in Malaysia at the population level, which provides important evidence for health policy development and planning. However, there are several limitations of this study. First, the precision of YLL estimates is entirely dependent on the quality of data on the underlying causes of death. NMCDs are not as reliable as MCD for reporting causes of death, which may lead to underestimation of causes of death. The recent implementation and widening coverage of verbal autopsy in Malaysia is expected to reduce the number of ill-defined causes of death. Furthermore, the findings described in the present study are limited to mortality and do not provide any information on the burden due to non-fatal health outcomes.

## Conclusions

Non-communicable diseases remain the largest contributors to premature mortality among older adults in Malaysia. Several national action plans are now underway in Malaysia with an aim to implement effective strategies to reduce the burden of NCDs. As the country undergoes demographic and epidemiological transition, sustained policy momentum and population awareness for NCD prevention and control is more important now than ever. Implementation of population-level NCD health promotion programmes, screening programmes among high-risk groups, and holistic intervention programmes among populations living with NCD are critical in reducing the overall burden of premature mortality.

## Data Availability

For data protection purposes, the data used for this study are not publicly available but are available from the Institute for Public Health, Ministry of Health Malaysia upon reasonable request and with permission from the Director General of Health Malaysia.

## Abbreviations

CSMF: Cause-specific mortality fractions
DALY: Disability-adjusted life year
DOSM: Department of Statistics Malaysia
GBD: Global Burden of Disease Study
MCD: Medically certified deaths
NCD: Non-communicable diseases
NMCD: Non-medically certified deaths
YLL: Years of Life Lost
